# Objective Bayesian fMRI analysis—a pilot study in different clinical environments

**DOI:** 10.3389/fnins.2015.00168

**Published:** 2015-05-12

**Authors:** Joerg Magerkurth, Laura Mancini, William Penny, Guillaume Flandin, John Ashburner, Caroline Micallef, Enrico De Vita, Pankaj Daga, Mark J. White, Craig Buckley, Adam K. Yamamoto, Sebastien Ourselin, Tarek Yousry, John S. Thornton, Nikolaus Weiskopf

**Affiliations:** ^1^Department for Brain Repair and Rehabilitation, UCL Institute of Neurology, University College LondonLondon, UK; ^2^Wellcome Trust Centre for Neuroimaging, UCL Institute of Neurology, University College LondonLondon, UK; ^3^Centre for Medical Image Computing, University College LondonLondon, UK; ^4^Siemens HealthcareCamberley, UK; ^5^Neuroradiological Academic Unit, Department of Brain Repair and Rehabilitation, UCL Institute of Neurology, University College LondonLondon, UK

**Keywords:** neurosurgical planning, passive fMRI, motor cortex, effect size, interventional MRI, bayesian statistics, false positive, false negative

## Abstract

Functional MRI (fMRI) used for neurosurgical planning delineates functionally eloquent brain areas by time-series analysis of task-induced BOLD signal changes. Commonly used frequentist statistics protect against false positive results based on a *p*-value threshold. In surgical planning, false negative results are equally if not more harmful, potentially masking true brain activity leading to erroneous resection of eloquent regions. Bayesian statistics provides an alternative framework, categorizing areas as activated, deactivated, non-activated or with low statistical confidence. This approach has not yet found wide clinical application partly due to the lack of a method to objectively define an effect size threshold. We implemented a Bayesian analysis framework for neurosurgical planning fMRI. It entails an automated effect-size threshold selection method for posterior probability maps accounting for inter-individual BOLD response differences, which was calibrated based on the frequentist results maps thresholded by two clinical experts. We compared Bayesian and frequentist analysis of passive-motor fMRI data from 10 healthy volunteers measured on a pre-operative 3T and an intra-operative 1.5T MRI scanner. As a clinical case study, we tested passive motor task activation in a brain tumor patient at 3T under clinical conditions. With our novel effect size threshold method, the Bayesian analysis revealed regions of all four categories in the 3T data. Activated region foci and extent were consistent with the frequentist analysis results. In the lower signal-to-noise ratio 1.5T intra-operative scanner data, Bayesian analysis provided improved brain-activation detection sensitivity compared with the frequentist analysis, albeit the spatial extents of the activations were smaller than at 3T. Bayesian analysis of fMRI data using operator-independent effect size threshold selection may improve the sensitivity and certainty of information available to guide neurosurgery.

## Introduction

Magnetic resonance imaging (MRI) is today commonly used in planning neurosurgical treatment, offering exquisite soft tissue contrast and geometric accuracy. A neurosurgical intervention, e.g., a brain tumor resection, has two principal aims: a maximal resection of the pathology with minimal damage to functionally important proximal brain structures. Modern MRI methods can support both goals. Standard structural MRI provides anatomical information about the tumor and surrounding brain tissue (Hall and Truwit, 2008; Wengenroth et al., 2011). Advanced MRI techniques, such as functional MRI (fMRI), yield otherwise unavailable spatial and functional localization of eloquent brain areas potentially invaluable in the neurosurgical planning stage (Wengenroth et al., 2011). Structural MRIs are routinely used for neuro-navigation during surgery.

Brain activity related to stimuli or tasks is localized by exploiting the blood oxygenation level dependent effect (BOLD effect). A per-voxel time-series statistical analysis estimates the temporal BOLD response amplitude identifying significantly task-related brain areas, the results being displayed as a statistical parametric map (SPM). Several methods have been established for this analysis. The most commonly used is the frequentist statistical approach based on a t-statistic (Friston, 2007), applying frequentist statistics to a general linear model (GLM) (Friston et al., 1994) describing the experimental paradigm. This approach rejects a given null hypothesis H0 (usually H0 = no activation present) at a pre-chosen level of significance (αvalue), which determines the level of false positives (Type I error) under a valid H0. In other words, the α-value determines the probability of labeling a region as active even though it is not. However, the frequentist statistic does not control directly for false negatives (Type II errors), i.e., that H0 is not rejected even though a region is in fact active. That is, the user is provided with no information regarding the probability of overlooking true brain activity. Despite this limitation frequentist statistics is widely used for analysis of clinical fMRI.

False positive and false negative results are a potential risk for the patient and must be controlled in planning surgical intervention (Bartsch et al., 2006; Haller and Bartsch, 2009). While false positive results compromise the extent of tumor resection if they obscure tumor tissue (Gorgolewski et al., 2012), false negative results may precipitate an overly aggressive resection by obscuring eloquent brain tissue (Haller and Bartsch, 2009). Two recent publications proposed solutions for this problem. Johnson et al. (2012) proposed a computationally demanding Bayesian statistics approach comprising of a mixed Gaussian model with different weighting of false negative and false positive findings via a loss function, demonstrated on a single tumor case. Durnez et al. (2013) proposed an advanced definition of the *p*-value based using frequentist statistics and independent component analysis, demonstrated in a group of five patients.

In this context Bayesian statistics have advantages over the frequentist approach, as this method does not suffer from multiple comparison limitations, and is not limited to controlling for false positives only. Bayesian statistics estimate the posterior probability of the effect given the fMRI data (Friston et al., 2002a,b). This probability is expected to be high in task-related brain regions and low in both brain areas not mediating performance of the task and in voxels suffering from poor data quality. Therefore Bayesian analysis can not only test for BOLD signal increases and decreases, but also detect non-responsive areas, and separate these from areas with unclear activation status which therefore provide statistical results with low-confidence. These results are encoded in posterior probability maps (PPMs) (Friston and Penny, 2003). This Bayesian approach is therefore particularly attractive for neurosurgical planning since it reveals explicitly both activated and non-activated regions. Moreover, the Bayesian approach could be particularly relevant for intra-operatively and post-surgically acquired fMRI data, as, during surgery both the functional localization and BOLD response could change due to tissue resection effects, e.g., active areas become non-activated or *vice versa* (Duffau, 2001). Such changes are not detectable via common frequentist analysis. Despite the advantages of Bayesian statistics for neurosurgery, clinical fMRI currently remains commonly analyzed using the frequentist techniques, since Bayesian methods are less widely known and practical implementation is computationally demanding.

A key benefit of Bayesian approaches is that they can automatically adapt to the spatial scale of signal in the data. One framework for doing this is to describe spatial dependencies using Markov Random Field priors (Gössl et al., 2001; Woolrich et al., 2004a). The original implementation of these algorithms is particularly computationally intensive, but this has since been ameliorated by the adoption of Variational Bayesian approaches (Celeux et al., 2003; Penny et al., 2005; Woolrich and Behrens, 2006). These approaches were generalized further using spatial Gaussian priors with the ability to model spatial non-stationaries (Harrison et al., 2007) and combine spatial and non-spatial priors (Groves et al., 2009). Alternative spatial models such as Bayesian wavelets (Flandin and Penny, 2007) and mixture models representing active and non-active voxels (Everitt and Bullmore, 1999; Hartvig and Jensen, 2000; Woolrich et al., 2005; Woolrich and Behrens, 2006) have also been proposed. Other authors proposed spatial non-stationarities not relying on Gaussian Markov Random Field priors permitting both non-stationary spatial and regressor-specific regularization (Vincent et al., 2010; Risser et al., 2011).

All Bayesian models provide posterior probability maps, which require thresholds to mask non-relevant brain activation and highlight significant activated regions. One of these thresholds, the “effect size threshold” (γ), is a potential obstacle to the practical use of Bayesian methods since it requires the definition of a minimal BOLD response effect size. In the presurgical environment this is the minimal BOLD response amplitude considered clinically meaningful. Obtaining an optimal and objective choice of this threshold is challenging, since the amplitude of the minimally expected BOLD response in any given situation is usually unknown and a subject-specific value. Furthermore, it may depend on several factors such as the paradigm, subject performance, physiological artifacts, and post processing steps. Thus, it is difficult to prescribe an *a priori* value. So far, no systematic method for determining a reasonable minimal effect size from a data set has been reported, and BOLD response amplitudes are rarely available in the literature: typical effect sizes for different paradigms, brain areas and populations including patients are not routinely reported. This may be viewed as a generic weakness of current brain imaging approaches, as effect sizes are routinely reported and discussed in many other scientific disciplines.

The aim of our study was to implement a complete Bayesian analysis framework for fMRI neurosurgical planning within a pipeline to allow automated on-site data processing. This required the development of a method for objective and algorithmic estimation of the effect size threshold, suitable for deployment in clinical applications. We developed and tested our framework in three parts. (1) Development—3T presurgical scanner: We developed the approach in healthy volunteers performing a passive motor paradigm in a standard 3T clinical MRI system. To calibrate the Bayesian analysis results we performed conventional frequentist inference analysis of the same data as reference. This was done for two surgical planning scenarios to determine the activation loci or the extent of the activated region. We developed a linear model to estimate the Bayesian effect size threshold from the individual volunteer data set. Compared to frequentist analysis, Bayesian fMRI analysis provided identification of activated, deactivated and non-activated brain regions. (2) Test—intra-operative 1.5T scanner: We measured the same healthy volunteers in a 1.5T intra-operative MRI scanner, to test our approach under technically challenging conditions. Compared with the 3T data, frequentist and Bayesian results provided less comprehensive information regarding the brain response to the task. (3) Clinical scenario—brain tumor patient: We tested the approach in a case study with a patient candidate for tumor resection. Bayesian statistics revealed similar activation patterns compared to the frequentist results and additional information about non-activated areas and areas with low statistical confidence.

## Materials and methods

### Ethics statement

The study was conducted with institutional research ethics committee approval of The National Hospital for Neurology and Neurosurgery and Institute of Neurology Joint Research Ethics Committee, ref 09/H0716/18. The healthy volunteers and the patient gave written consent to their participation after receiving oral and written information as required and approved by the research ethics committee.

### Development—3T presurgical scanner

#### Volunteers

Ten healthy right-handed volunteers participated in the study (seven males, age = 34.8 ± 6.2 years [mean ± standard deviation]).

#### Equipment

Each volunteer was examined in a standard clinical radiology suite with a 3T MAGNETOM Trio TIM system whole-body MRI (Siemens Healthcare, Erlangen, Germany) equipped with the manufacturer's 32-channel RF head coil. The data were processed on a computer with 16 3.4 GHz AMD Opteron (tm) processor cores with 64 GB RAM and Debian Linux “squeeze” version 6.0.7.

#### Data acquisition

For fMRI, a time series of 142 echo-planar images (EPIs) was acquired with axial oblique orientation aligned to the anterior commissure to posterior commissure line covering the whole brain using repetition time TR|echo time TE|flip|angle α = 2260 ms|30 ms|90°, field of view FoV|matrix|phase-encoding = 192 × 192 mm^2^|64 × 64|AP, slices|thickness|gap = 42|2.7 mm|12%, bandwidth BW|echospacing = 2112 Hz/Px|0.56 ms, grappa-factor|reference lines = 2|24, volumes|duration = 142|5:28 min. A map of the static magnetic field (B_0_) using a double gradient-echo sequence was acquired for correction of susceptibility-induced geometric distortion of the EPI images (Andersson et al., 2001; Hutton et al., 2002) with the following parameters: TR|TE1|TE2|α = 688 ms|4.92 ms|7.38 ms|60°, FoV|matrix = 192 × 192 mm^2^|96 × 96, slices|thickness|gap = 42|3 mm|0%, BW = 259 Hz/Px, duration = 2:13 min. Anatomical images were acquired using a 3D T1-weighted MPRAGE (magnetization prepared rapid gradient echo Mugler and Brookeman, 1990) sequence with the following parameters: TR|TE|TI|α = 2200 ms|2.88 ms|900 ms|10°, FoV|matrix = 220 × 220 × 203 mm^3^|192 × 192 × 176, BW = 240 Hz/Px, duration = 7:02 min.

#### fMRI of passive hand motion

A passive motor paradigm with simple cueing was used, chosen for operational simplicity and applicability in motor functionally impaired or anaesthetized patients. An operator standing next to the scanner bed flexed the volunteer's upwards-facing right hand fingers toward the palm and then extended them back. Operators were trained to flex with a frequency of 1–1.5 s. Stimulation consisted of a passive hand motion block of 16 s duration followed by 16 s rest. The stimulation was repeated 10 times. The operator was visually cued to start and stop the movements in synchrony with the acquisitions but did not receive any other prompts such as prompts for the frequency of flexing.

#### Data processing

The data sets were individually processed for each volunteer. The analysis pipeline was automated using a custom-written toolbox MRIST (MR Imaging and Spectroscopy Toolbox^1^), which combines tools mainly from SPM12b^2^, FSL 5.0.1^3^, MATLAB^4^ (R2012a, 1984–2012 The Math Works, Inc.) and BASH into a Debian Linux “squeeze” version 6.0.7^5^ command line based data analysis pipeline.

Pre-processing was performed with SPM12b and MATLAB after DICOM to NIfTI image file format conversion. Functional EP images were corrected for susceptibility-related distortion based on a voxel displacement map (in the phase encoding direction) determined from the B_0_ fieldmap. Motion correction also included correction for static susceptibility-related distortions and correction for the interaction between head orientation and B_0_ distortion, i.e., dynamic susceptibility-related distortion effects (Andersson et al., 2001; Hutton et al., 2004). Finally, the distortion corrected images were smoothed with an isotropic Gaussian kernel with a full width at half maximum (*FWHM*) of 4 mm. The smoothed images were used in the next step for both frequentist and Bayesian frameworks, to ensure equal pre-processing steps for both frameworks.

For the reference frequentist and Bayesian statistical analyses, the smoothed data were modeled using a general linear model (GLM) comprising two groups of regressors modeling different aspects of the *in vivo* situation. The first group modeled the task-related BOLD response. It was constructed by convolving the stimulus function (boxcar function describing the block design) with the canonical hemodynamic response function as implemented in SPM (Friston, 2007). To accommodate for variability in the hemodynamic response a regressor for the temporal derivative of the canonical response (Friston, 2007) was also added. The second group included the six rigid-body movement parameters estimated in the motion correction step to account for potential motion induced artifacts. Two t-contrasts were tested in the frequentist analysis. One tested for a significant positive BOLD response modeled by the task block indicator convolved with the hemodynamic response function (HRF). The second one tested for a negative response.

For the Bayesian analysis^6^ additional Bayesian-specific SPM12 analysis parameters were selected: Unweighted Graph-Laplacian (UGL) for signal and noise priors comprising local adaptive smoothing, and an autoregressive model of order two for the model of serial correlation of the noise. The priors described in Penny et al. (2005) allow for the prior spatial smoothness to be different for each regression coefficient. This makes sense, as different experimental effects are likely to exist at different spatial scales. Moreover, because these smoothness parameters are estimated from the data (in an empirical Bayes optimization scheme) the final estimated parameters are in this sense rather insensitive to the prior. However, it is nonetheless assumed that the prior smoothness for each regression coefficient does not vary over space. One can relax this assumption by using a prior based on a Weighted Graph Laplacian (Harrison et al., 2007). This has the advantage of preserving edges in functional activation maps but the disadvantage of increased computational complexity and was not applied here.

In the following*N*(*x*; *m*, *C*) denotes a multivariate normal distribution of random variable *x* having mean *m* and covariance *C*. The algorithm fits a GLM to fMRI data according to

(1) $$y_{i}=X\beta_{i}+e_{i}$$

where *y*_*i*_ is the fMRI time series at voxel *i*, *X* the design matrix, β_*i*_ are the unknown regression coefficients and *e*_*i*_ is the error time series. The Bayesian algorithm then estimates a posterior distribution over regression coefficients

(2) $$p\left(\beta_{i}|Y\right)=N(\beta_{i};{\hat{\beta }}_{i},{\hat{\Sigma }}_{i})$$

as described in Penny et al. (2005), where ${\hat{\beta }}_{i}$ is the posterior mean, ${\hat{\Sigma }}_{i}$ is the posterior covariance and *Y* denotes fMRI data over all voxels. Regression coefficients at a given voxel are (softly) constrained to be similar to those at nearby voxels. The strength of this constraint is determined by a spatial precision parameter that is estimated from the data. Different regression coefficients have different spatial precisions allowing each putative experimental effect to have its own spatial regularity. Contrasts are then used to test for specific effects

(3) $$a_{i}=c^{T}\beta_{i}$$

where *a*_*i*_ is the effect size at voxel *i*, and *c* is the contrast vector used to test for that effect. This gives rise to a posterior distribution over effect size

(4) $$p\left(a_{i}|Y\right)=N(a_{i};\mu_{i},\sigma_{i})$$

where

(5) $$\mu_{i}=c^{T}{\hat{\beta }}_{i}\text{ and }\sigma_{i}^{2}=c^{T}{\hat{\Sigma }}_{i}c.$$

The effect sizes reported in this paper are expressed in percentage of signal change (task regressor in SPM) compared to the temporal mean (mean regressor in SPM) in each voxel. Thus an effect size of 1.5 is a 1.5% increase in local activity. We used a contrast to test for positive BOLD responses modeled by the task stimulus function.

**Figure 1:**
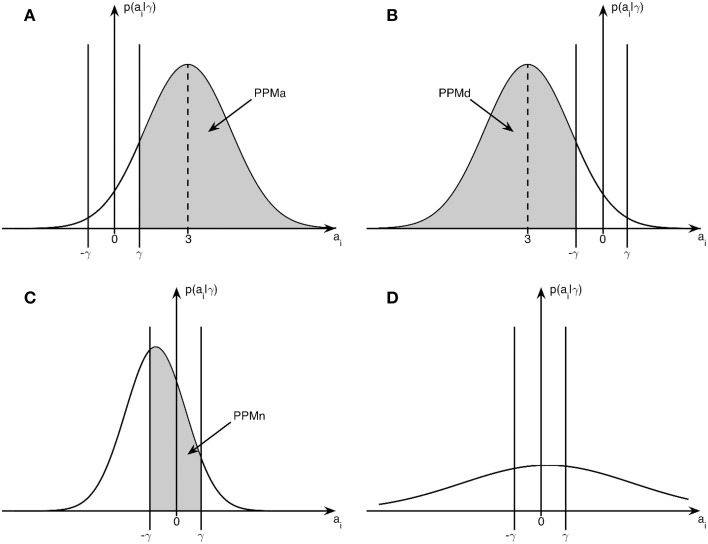
**Posterior distribution and effect size: example posterior distributions from Bayesian inference in relation to the effect size threshold γ. (A)** Posterior distribution for an activated voxel with probability PPMa, **(B)** Posterior distribution for a deactivated voxel with probability PPMd, **(C)** Posterior distribution for a non-activated voxel with probability PPMn, **(D)** Posterior distribution for a voxel with low-confidence.

The probabilities of activation (PPMa), deactivation (PPMd), and non-activation (PPMn), are given by:

(6) PPMa=1−Ncdf(γ;μ,σ)

(7) PPMd=Ncdf(−γ;μ,σ)

(8) PPMn=1 − PPMa − PPMd

where Ncdf(γ; μ, σ) denotes the cumulative density function for a univariate Gaussian with mean μ, standard deviation σ, evaluated at γ. Graphically illustrated, PPMa corresponds to the shaded area in Figure 1A, PPMd corresponds to the shaded area in 1b and PPMn to the shaded area in 1c. Subfigure 1d illustrates the case with low-confidence where none of PPMa, PPMd, and PPMn lead to significant classification of a voxel.

In addition to the specification of an effect size threshold γ, the Bayesian PPM approach (Friston and Penny, 2003) also requires a threshold on the posterior probability itself. Voxels with posterior probability greater than this threshold will appear in the relevant PPM. We denote this threshold as *p*_*T*_. Once the voxel probabilities have been computed they can then be assigned to one of four categories as follows: voxels for which PPMa > P_T_ are classified as activated, voxels for which PPMd > P_T_ are classified as deactivated, voxels for which PPMn > P_T_ are classified as non-activated, and voxels categorized as “low-confidence.” If a voxel is “activated” we are confident the effect is positive; if it is “deactivated” we are confident it is negative; and if it is “non-activated” we are confident it is around zero. If none of the above criteria are met the voxel is assigned to the “low-confidence” category. It is also convenient to express this probability in the form of a log odds ratio threshold or log Bayes Factor Threshold (*LBT*) where

(9) $$LBT=log\frac{p_{T}}{1-p_{T}}$$

For example, *p*_*T*_ = 0.95 corresponds to *LBT* = 3, and p_T_ = 4.5 × 10^−5^ corresponds to *LBT* = 10. A value of *LBT* = 10 is commonly used in Bayesian neuroimaging to determine relevant effects (Penny and Ridgway, 2013).

#### Effect size threshold (γ) for the bayesian analysis

In order to estimate the above posterior probability maps, the Bayesian analysis requires a threshold for the effect size threshold γ (Figure 1). We defined γ as a certain percentage of the median BOLD amplitude of the voxels with the top 0.1% positive BOLD response of the whole brain, based on the rationale that maximal and minimal relevant BOLD responses are tightly coupled and that γ is the same throughout the brain. Since it is not known from the literature whether this is a reliable estimate of the minimal γ, or what percentage of the peak BOLD amplitude should be chosen, we tested and cross-calibrated the approach based on clinical best judgment. In current practice the best available clinical fMRI evaluation is based on frequentist statistics and expert judgment (FitzGerald et al., 1997; Rutten et al., 2002). Here, a clinically knowledgeable expert observer determines a *p*-value or *t*-value threshold for the SPM taking into account the clinical purpose, paradigm, localization, background noise, amplitude, and extent of activation, in order to achieve an optimally plausible estimate of the activated motor and somatosensory cortex.

In the present study two expert observers (TY, LM) with 19 and 7 years neuroradiological fMRI experience defined, in consensus, for each volunteer, two clinically plausible *t*-value thresholds α for the positive BOLD response of the primary motor cortex on the SPM t-map from the 3T pre-operative scanner. The first *t*-value threshold *t_*loc*_* was chosen to most sensitively indicate the activation loci (with the criteria that activations should be limited to only pre- and post-central gyri regions and not extend further) and the second *t_*ex*_* to visualize the extent of the activation area (defined as the maximum extent of plausible activation without apparent activation in the white matter). Both criteria were based on two concepts: first, identifying the primary motor cortex with anatomical landmarks (Yousry et al., 1997); second, using local established experience of (pragmatically) thresholding clinical fMRI for presurgical planning, as no published and generally accepted standard methods exist. For this purpose t-maps up-sampled to 1 mm^3^ resolution using a 7th order spline interpolation method in SPM12b were displayed overlaid on an equivalent up-sampled anatomical scan obtained in the same scanning session having the same resolution. The resulting thresholds served in the following analyses as the reference for choosing two effect size thresholds required for the above described Bayesian analysis: activation loci γ_*loc*_ and the extent γ_*ex*_. In the further processing steps only these areas in the motor cortex were considered and other activated areas were neglected, but not classified further, e.g., “not-active.”

For cross-calibration of the standard frequentist and the Bayesian approaches, we used a custom-written MATLAB script to estimate γ for the corresponding Bayesian PPM resulting in a best match between the SPM calibrated by the clinical expert observers and PPM using the Jaccard index (Jaccard, 1901) as the maximal overlap measure. The calibration was based on a binary reference map of the right hand motor cortex based on the expert observer selected thresholds: A cluster map with connectivity^7^ 18 was generated from each low resolution SPM, thresholded using one of the observer-determined values *t_*loc*_*, *t_*ex*_*. All clusters except for the right hand motor cortex cluster *C_*Ref*_* were erased manually from the map. For comparison the PPMs were clustered with the same algorithm, but without erasing any clusters. These clustered PPMs were voxel-wise compared with the respective *C_*Ref*_*: All clusters *i* in the PPM overlapping the motor cortex reference *C_*Ref*_* were selected $C_{Bayes}=\;\cup_{i}\left\{C_{Bayes,i}\cap C_{REF}>\{\}\right\}$ and the overlap between *C_*Ref*_* and C_Bayes_ was measured with the Jaccard index *J*

(10) $$J\left(C_{Ref},C_{Bayes}\right)=\;\frac{\left|C_{Ref}\cap C_{Bayes}\right|}{\left|C_{Ref}\cup C_{Bayes}\right|}$$

To estimate the effect size with the best overlap the procedure was repeated with different effect sizes from γ = 0 to γ_*max*_ where no activation was present (in the individual volunteer) using an upper threshold of *LBT* = 10 and an effect size increment of Δγ = 0.01. All Jaccard indices were plotted against γ and smoothed with a box kernel of eight. The γ with the highest Jaccard index was identified as the optimal effect size threshold yielding the best match of the SPM and PPM activation extents. The procedure was performed individually for *t_*loc*_* and *t_*ex*_*.

We tested whether γ_*loc*_ and γ_*ex*_ could be predicted from the percentage of the top 0.1% positive BOLD responses by robust regression analysis. With the rationale that a region with no brain activation does not show a BOLD effect, and hence has an effect size of zero, an intercept-free bisquare-weighted robust regression analysis (Mohammadi et al., 2013) was implemented in MATLAB. We used the same model parameters for automatically determining the effect size thresholds for the data recorded on the intra-operative scanner since our method is based on the data itself and therefore was presumed generally applicable. For the regression as well as for the later data analysis, the brain was masked with a binary image derived from tissue segmentation of the anatomy to exclude unwanted apparent activation in non-brain regions.

#### Result maps

The 3T frequentist result maps (SPMs) were thresholded based upon volunteer-specific *t*-values determined by the two clinical experts for the 3T data. The Bayesian results (PPMs) are based on the effect size threshold provided by the linear regression detailed in the previous section, and using *LBT* = 10. To compare the 1.5T and 3T scanner data all statistical maps were registered and resliced to the AC-PC reoriented intra-operative scanner anatomical reference images (acquired in the second part “test—intra-operative 1.5T scanner”) using 7th order spline interpolation to 1 mm^3^. SPMs were plotted for all volunteers with three orthogonal slices intersecting the center of mass of the *t_*loc*_* thresholded t-map from the frequentist analysis of the 3T scanner data located in the right hand motor area. For comparing results, the experienced observer-defined t-thresholds were converted into a familywise error corrected *p*-value, since this metric is more commonly reported in the literature. The Bayesian analysis labeled voxels as activated for a positive BOLD response, deactivated for a negative BOLD response, and non-activated for no relevant changes in the BOLD signal. For easier volumetric comparison of the activated region we included the cluster volumes [mm^3^] for frequentist analysis (CSF) and Bayesian analysis (CSB) in **Figures 3, 4**.

#### Quantitative cluster analysis

To support the visual assessment of the result maps, a quantitative cluster analysis of the activated motor cluster of the spatially up-sampled Bayesian maps was performed. The results were compared with the up-sampled frequentist maps as reference. We counted true positive (TP), true negative (TN), false positive (FP) and false negative (FN) voxels in the masked brain. We calculated the sensitivity = TP / (TP + FN) and false discovery rate (FDR) = FP / (FP + TP). The FDR (Benjamini and Hochberg, 1995) was chosen in preference to the specificity = TN / (TN + FP) as the large proportion of true negative voxels (TN >> TP) led to a specificity ~1.

### Test–intra-operative 1.5T scanner

For purposes of comparison we measured the same volunteer group in a 1.5T intra-operative scanner with a dedicated surgery head coil. While this system opens up the possibility of intra-operative fMRI (ifMRI), coil and scanner specifications in this case provide further technical challenges, in particular a low signal-to-noise ratio (SNR) profile and nonlinear distortions due to the short gradient system. Currently the scanner is used to obtain pre- and intra-operative standard anatomical imaging to guide neurosurgery and the potential use of intra-operative fMRI has not been explored so far at our institution. Hence this study section was a pilot with the aim of testing our methods under instrumentally challenging, but otherwise physiologically optimal conditions (healthy subjects, no anesthesia, no craniotomy), as a first step to the future implementation of ifMRI.

#### Equipment

Each volunteer was examined in an intra-operative 1.5T MAGNETOM Espree TIM system whole-body MRI (Siemens Healthcare, Erlangen, Germany) located in a neurosurgical theater. This scanner was equipped with a dedicated 8-channel receive-only surgical head coil (NORAS MRI products GmbH, Hoechberg, Germany). For safety reasons, the head-holder pins of the 8-channel coil, designed for surgical (invasive) head fixation were replaced by a foam pad for these experiments. The scans were performed approximately concurrently with the 3T scans: For five volunteers the time interval between scans was 1 week; for the remaining volunteers the intervals were 0, 2, or 10 weeks.

#### Data acquisition

For fMRI, a time series of 142 echo-planar images (EPIs) were acquired with axial oblique orientation aligned to the anterior commissure to posterior commissure line covering the whole brain using TR|TE|α = 3100 ms|40 ms|90°, FoV|matrix|phase-encoding = 192 × 192 mm^2^|64 × 64|AP, slices|thickness|gap = 42|2.7 mm|12%, BW|echospacing = 1446 Hz/Px|0.82 ms, grappa-factor|reflines = 2|24, volumes|duration = 104|5:32 min. A map of the static magnetic field (B_0_) using a double gradient-echo sequence was acquired for correction of susceptibility-induced geometric distortion of the EPI images (Hutton et al., 2002, 2012) with the following parameters: TR|TE1|TE2|α = 630 ms|4.92 ms|9.68 ms|60°, FoV|matrix = 192 × 192 mm^2^|64 × 64, slices|thickness = 42|3 mm, BW = 260 Hz/Px, duration = 1:23 min. Anatomical images were acquired using a T1-weighted MPRAGE (magnetization prepared rapid gradient echo Mugler and Brookeman, 1990) sequence and the following parameters: TR|TE|TI|α = 2250 ms|3.70 ms|1100 ms|15°, FoV|matrix = 220 × 220 × 221 mm^3^|192 × 192 × 192, BW = 180 Hz/Px, duration = 7:14 min.

#### Data processing

The data pre-processing was similar to that used for the 3T data. An additional step was included to correct for the image distortions originating from the relative non-linearity of the imaging gradients resulting from engineering limitations of the short and wide intra-operative scanner magnet bore. This problem was exacerbated by the necessity to scan with an off-centered head position (as is routine practice in our iMRI practice) due to restrictions of the intra-operative head-clamp set-up: the head (patient orientation: head first supine) was shifted 60 mm from isocentre along the anterior direction (positive y-axis of the scanner) out of the sphere of optimal gradient linearity (*d*~120 mm). The non-linearity correction was applied to the functional and the structural NIfTI images as the first processing step, and to the voxel displacement map estimated using the B_0_ fieldmap. The correction was implemented in MATLAB using the manufacturer's spherical harmonic description of the imaging gradient field non-linearity and a correction method published in Janke et al. (2004). The underlying image resampling was done with SPM12b using non-linear three-dimensional image deformations and a 7th order spline-based interpolation.

#### fMRI analysis and result maps

The smoothing and statistical processing with frequentist and Bayesian modeling were kept identical as for the 3T data (see first part “development—3T presurgical scanner”). The threshold process was adapted to cope with the limited SNR of the surgery head coil. An initial frequentist statistical analysis of the 1.5T functional data revealed that the activation extent was constrained to the center of the motor region in all volunteers. Thus a comparison of our Bayesian model with expert observer thresholds was not possible and a fixed familywise error corrected *p*-value of *p*(FWE) = 0.05 was chosen.

In contrast to frequentist statistics we could apply our proposed effect-size model for motor loci and motor extent, as the Bayesian analysis applies additional data-driven smoothing increasing the effective Signal-to-Noise.

We generated the presented high-resolution result maps with the same procedure as in the first part “Development—3T presurgical scanner.”

#### Quantitative cluster analysis

To support the visual assessment of the result maps, a quantitative cluster analysis was performed in the same way as described in the part “development—3T presurgical scanner.”

### Clinical scenario–brain tumor patient

We tested our Bayesian analysis framework on data obtained from a 58y male patient with an oligodendroglioma in the left inferior precentral gyrus. The patient was suffering from speech disturbance, but had no loss of motor functions and was receiving no medication at the time of the study.

#### Data acquisition

Echo-planar images and fieldmaps were acquired as described in the first part “development—3T presurgical scanner.” Anatomical images were also acquired identically except for a slice thickness of 1.1 mm. For diagnostic imaging of the tumor a sagittal fluid attenuated inversion recovery (FLAIR) image was acquired using the following parameters: TR|TE|TI|α = 6000 ms|388 ms|2200 ms|120°, FoV|matrix = 250 × 250 × 160 mm^3^|256 × 256 × 160, slices|thickness = 160|1.0 mm, BW = 240 Hz/Px, slice-partial-fourier = 7/8 grappa-factor|reference lines = 2|24, duration=7:02 min.

#### Data processing, fMRI analysis, and result maps

The data preprocessing and statistical modeling were identical to the first part “development—3T presurgical scanner.” We used our effect size threshold model to generate Bayesian result maps for the motor localization (γ_*loc*_) and the motor extent (γ_*ex*_). To compare these results with a frequentist statistics reference, one clinical expert (LM) selected respective thresholds for motor localization (t*_*loc*_*) and motor extent (t_*ex*_) on the t-map from the frequentist statistics. Based on these references we estimated the best fitting effect size threshold as described in the first part “development—3T presurgical scanner” and compared these effect sizes with γ_*loc*_ and γ_*ex*_ estimated by our model.

For the results all fMRI result maps and the FLAIR images were registered to the AC-PC oriented structural data set and up-sampled to 1 mm^3^ resolution using 7th order interpolation.

## Results

### Development—3T presurgical scanner

#### Estimating the effect size threshold

**Figure 2:**
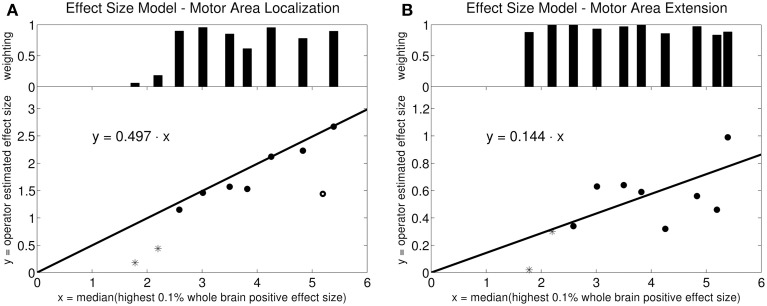
**Linear model for effect size estimation: Intercept-free robust linear regression of the estimated effect size against the median effect size of the 0.1% highest activation-signal amplitude voxels of the whole brain**. The weighting on each data point in the robust fit is plotted in the bar diagram at the top. **(A)** Motor area localization: one point was weighted with zero by the robust fit algorithm (circle). The other points were weighted between 0.06 and 1.0. The starred points mark the two outliers in the data set (volunteers 9 and 10). **(B)** Motor area extension: the points were weighted between 0.84 and 1.0. The robust algorithm did not exclude any points as outliers. The starred points mark the two outliers in the data set (volunteers 9 and 10).

The robust fit revealed a slope of 0.497 for the activation loci effect size γ_*loc*_ using an intercept-free regression of the median of the top 0.1% positive BOLD response amplitudes against the expert-defined γ as independent variable (Figure 2A). The fit algorithm excluded one outlying point (circle at x = 5.2). All remaining points were weighted between 0.06 and 1.0. For activation extent effect size γ_*ex*_, the same robust fit revealed a slope of 0.144. No points were excluded. The weights were between 0.84 and 1.0 (Figure 2B).

#### fMRI result maps

**Figure 3:**
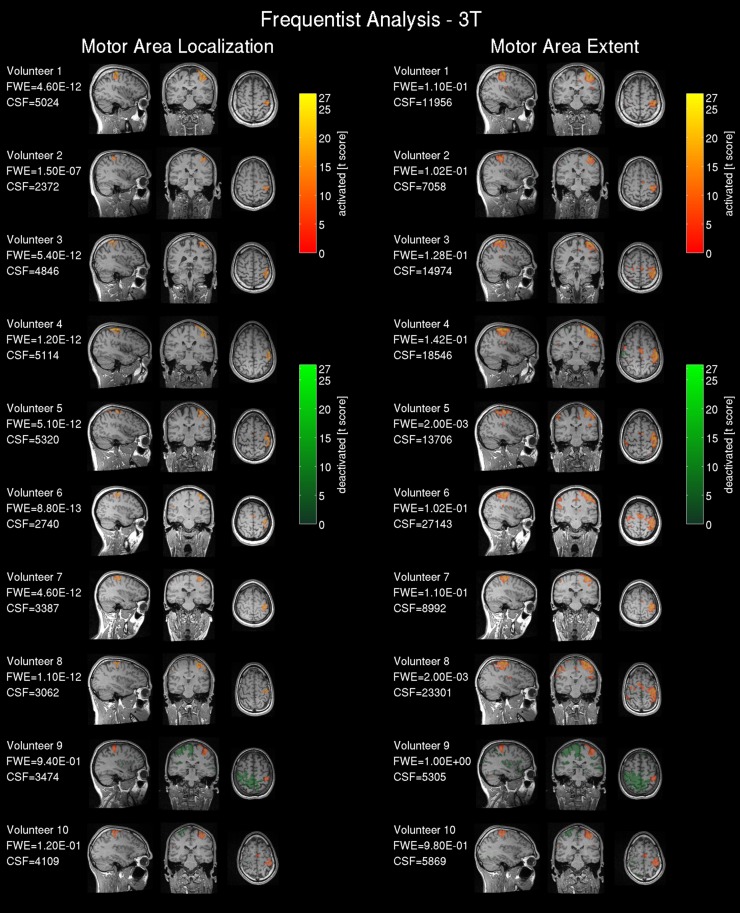
**Frequentist analysis for the 3T pre-operative scanner: The left side shows the activity maps using the operator selected t-threshold revealing the central motor area location; on the right side the operator selected t-threshold approximating the motor area extent**. The maps are labeled as activated=positive BOLD response, deactivated=negative BOLD response. The used familywise error (FWE) threshold is the FWE-value converted from the t-threshold estimated by the operators. The cluster size of the frequentist analysis (CSF) is displayed in number of voxels.

**Figure 4:**
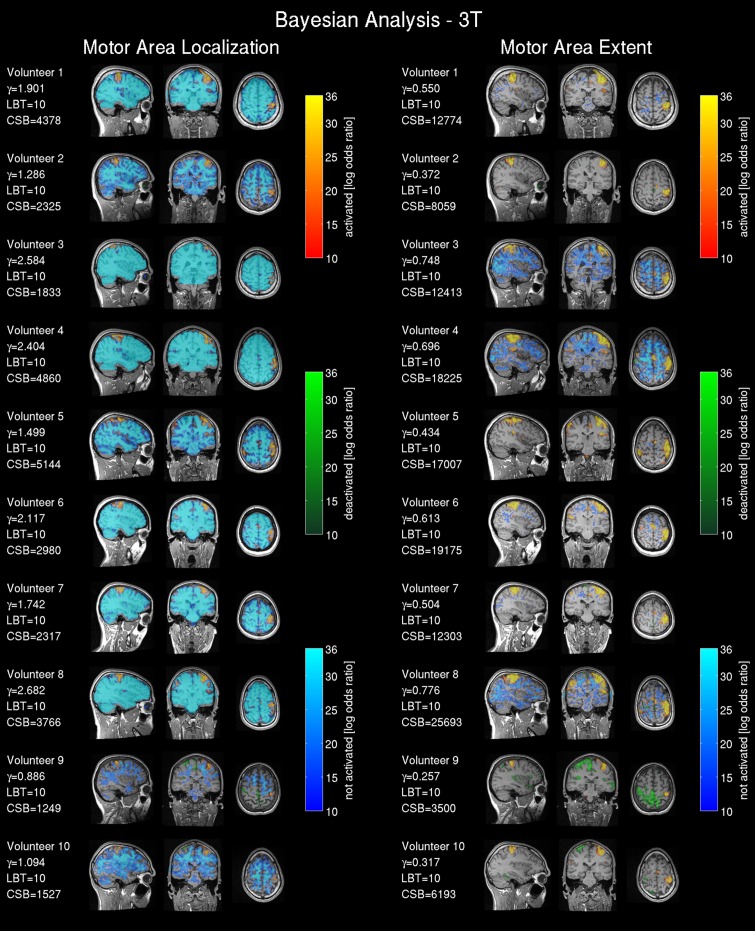
**Bayesian analysis for the 3T pre-operative scanner: Log Bayes factor maps showing the activation pattern and strength expressed by the voxel-wise log Bayes factor**. The left side shows the activity maps using the effect size threshold γ_*loc*_ revealing the central motor area localization; on the right side the effect size threshold γ_*ex*_ revealing the motor area extent. The maps are labeled as activated=positive BOLD response, deactivated=negative BOLD response and non-activated = no changes in the BOLD contrast, non-colored = low-confidence, i.e., BOLD activation status cannot be determined based on data. The effect size threshold (γ) calculated with the proposed linear model and the cluster size in voxels extracted from the Bayesian analysis (CSB) are displayed. Results from volunteers 9 and 10 are considered as outliers due to data quality problems.

Results from the 3T scanner revealed activation in the left hemispheric motor and somatosensory cortex corresponding to the right hand in all volunteers in agreement with the literature (Lotze et al., 1999) for frequentist (Figure 3) and Bayesian analysis (Figure 4). Compared with the frequentist analysis the Bayesian analysis showed similar activation patterns (yellow/red) in seven of 10 volunteers using the automatically determined γ_*loc*_ and similar patterns in all volunteers for γ_*ex*_. The somatosensory cortex was conspicuously visible using the γ_*ex*_ for frequentist (Figure 3, right) and Bayesian analysis (Figure 4, right) and less prominent in the γ_*loc*_ threshold results (Figure 3, left and Figure 4 left). Volunteer 9 showed conspicuous deactivation on the hemisphere ipsilateral to the movement and volunteer 10 small deactivated clusters for the γ_*ex*_ thresholded PPM. For those volunteers the clinical observers chose a low t-threshold leading to a high familywise error-corrected *p*-value above the accepted value of *p* = 0.05. Hence both data sets may be considered as outliers.

The non-activated areas (blue) revealed by the Bayesian analysis using γ_*loc*_ (Figure 4, left) were pronounced and tightly encapsulated the activated areas in most of the volunteers except volunteers 9 and 10. Volunteer 2 showed a weaker and less-pronounced probability of non-activated brain regions. The extent of the non-activated areas based on γ_*ex*_ were overall reduced (Figure 4, left) compared with the γ_*loc*_ based results and pronounced only in three volunteers. Non-colored regions in the brain correspond to the low-confidence category (neither activated, deactivated or non-activated).

#### Quantitative cluster analysis

**Figure 5:**
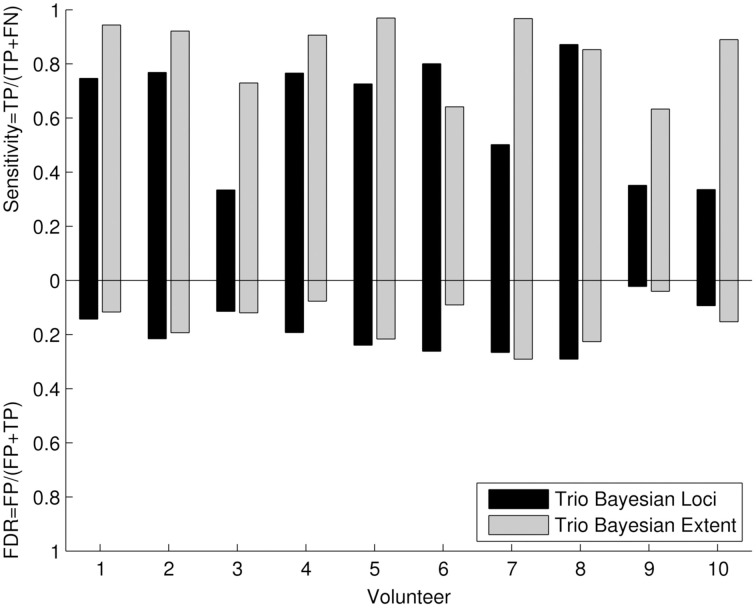
**Quantitative cluster analysis of the 3T Bayesian results: The quantitative cluster analysis reveals sensitivity and false discovery rate (FDR) for the 3T Bayesian results**. The activated motor clusters using the 3T frequentist result maps as reference.

The quantitative cluster analysis reflected the results of visual comparison between frequentist and Bayesian results. Six out of Ten volunteers showed high sensitivity (Figure 5) for revealing the motor loci with the new Bayesian framework. For the remaining four volunteers, a lower sensitivity was observed in two volunteers (three and seven) due to the underestimation of the motor loci cluster size and a data quality problem in volunteers 9 and 10. The sensitivity for revealing the motor extent was higher than the sensitivity for revealing the motor loci in all volunteers. False discovery rates were low for all assessments.

### Test—intra-operative 1.5T scanner

#### fMRI result maps

**Figure 6:**
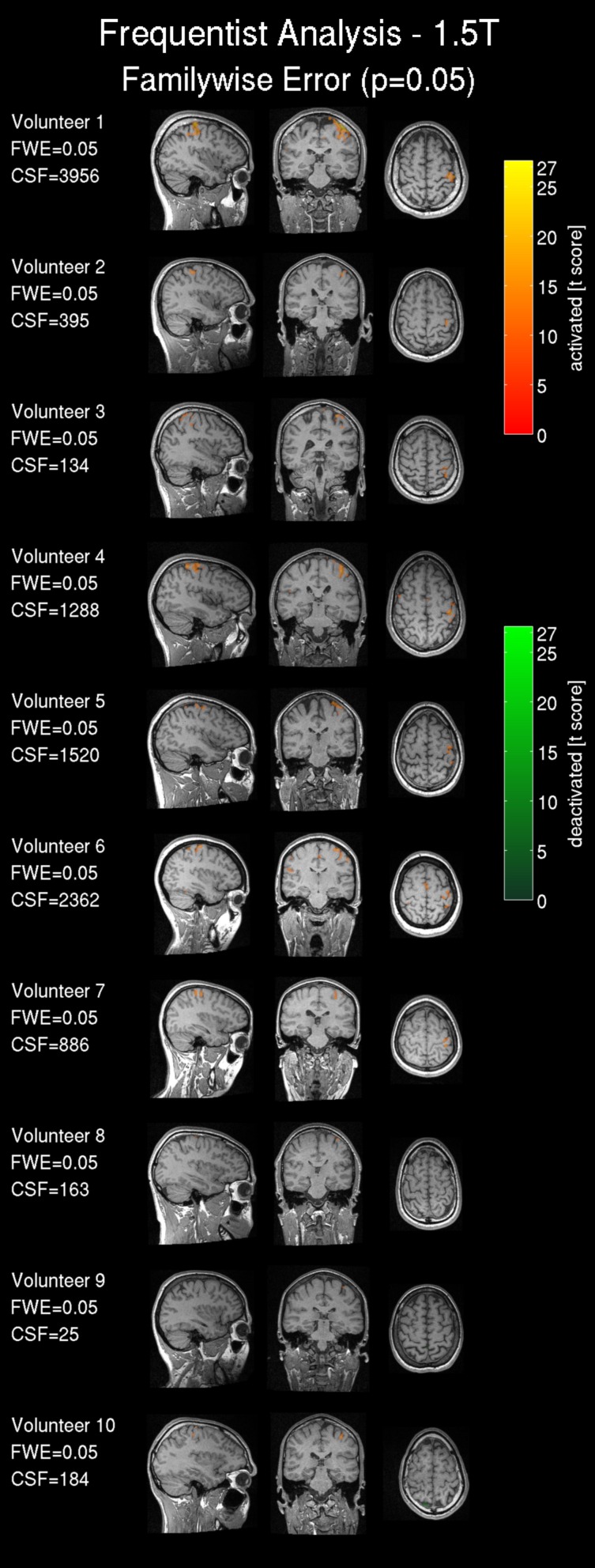
**Frequentist analysis for the 1.5T intra-operative scanner (similarly labeled to Figure 3): Frequentist analysis showing *t*-values of significant active areas using a familywise error (FWE) threshold *p* = 0.05**. The cluster size of the frequentist analysis (CSF) is displayed in number of voxels.

**Figure 7:**
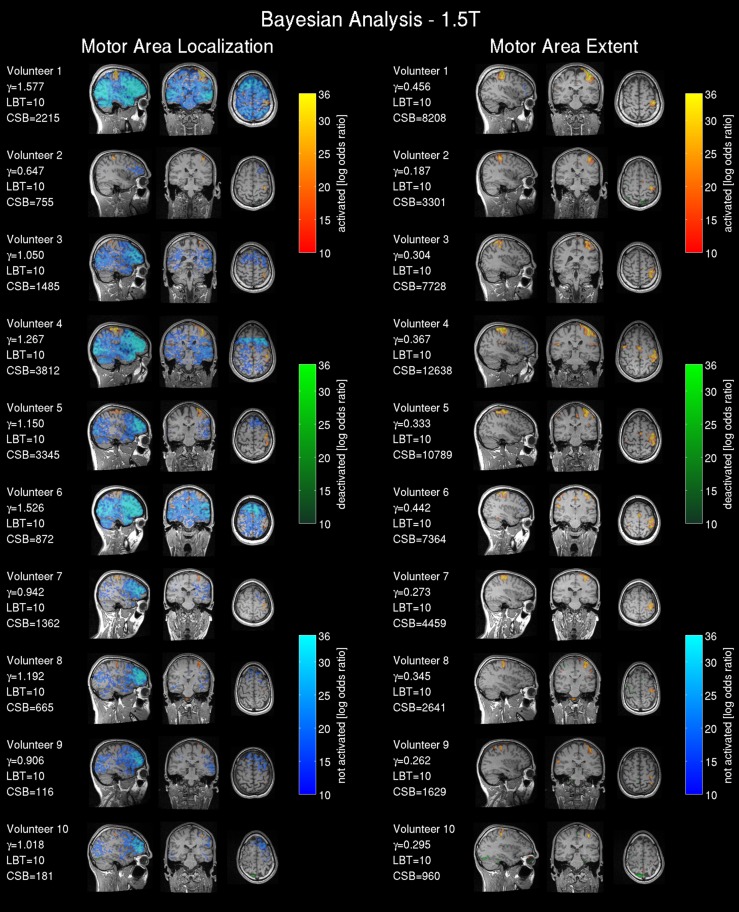
**Bayesian analysis for the 1.5T intra-operative scanner (similarly labeled to Figure 4): Bayesian analysis log Bayes factor maps showing the activation pattern and strength expressed by the voxel-wise log Bayes factor**. The effect size threshold (γ) calculated with the proposed linear effect size model and the cluster size in voxels extracted from the Bayesian analysis (CSB) are displayed.

Frequentist analysis results from the 1.5T intra-operative scanner revealed overall less prominent activation patterns (Figure 6) compared with the 3T data. Only volunteers 1, 4, 5 and 6 showed a somewhat pronounced activation response compared with other 1.5T maps. Most of the 1.5T Bayesian analysis maps (Figure 7) showed more widespread activation patterns than the frequentist maps, but these were still less conspicuous than the equivalent 3T results (Figures 3, 4); only volunteers one, four, and five showed a larger activation area in the frequentist and Bayesian maps. Overall the reduced cluster sizes *CSF* and *CSB* at 1.5T corroborated this visual assessment. For the activation loci base effect size γ_*loc*_ a marked reduction in the probability for non-activated areas was observed in all images. Except for volunteers one, four, and six the majority of the voxels could not be classified as activated, deactivated, or not active and were marked as low-confidence. The extents of the non-activated areas based on γ_*ex*_ were overall further reduced (Figure 6, right) to isolated small clusters in some volunteers. Compared to the 3T data, at 1.5T all frequentist and Bayesian maps provided less comprehensive information regarding the brain response to the task.

#### Quantitative cluster analysis

**Figure 8:**
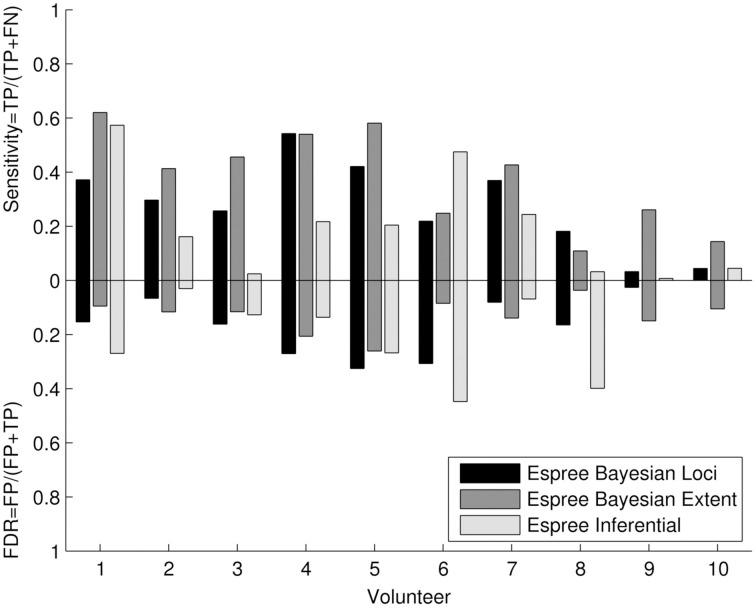
**Quantitative cluster analysis of the 1.5T Bayesian results: The quantitative cluster analysis reveals sensitivity and false discovery rate (FDR) for the 1.5T Bayesian results and frequentist results of the activated motor clusters using the 3T frequentist result maps as reference**.

The quantitative cluster analysis (Figure 8) reflects the visual assessment between frequentist and Bayesian results. Bayesian and frequentist statistics showed generally lower sensitivity compared with the 3T data (Figure 5), but a higher sensitivity for Bayesian analysis compared with the 1.5T frequentist results. The results from volunteers 9 and 10 revealed a similar data quality problem as seen at 3T. False discovery rates were low to moderate.

### Results: tumor case

**Figure 9:**
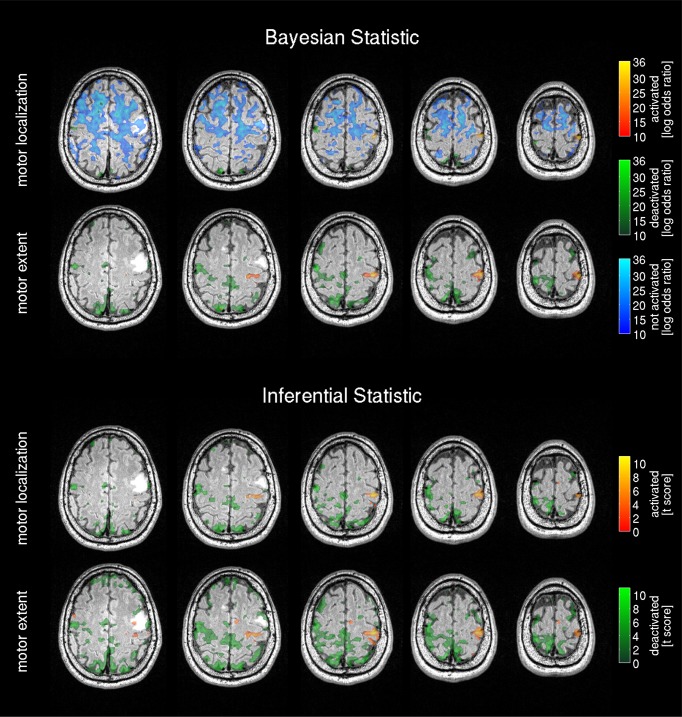
**Bayesian analysis and frequentist analysis results of the patient data: The Bayesian analysis for the tumor patient data shows the activation pattern and strength expressed by the voxel-wise log Bayes factor (similarly labeled to Figure 4)**. The activated motor region matches the respective area in the frequentist statistics results.

Both threshold models for the Bayesian effect size showed prominent activation in the right hand motor cortex for motor localization and motor extent (Figure 9). The activated region was not infiltrated by MRI-visible tumor tissue (hyper-intense region), but slightly dislocated posteriorly by the space-occupying lesion. Similarly to the healthy subjects' 3T results, the motor location threshold revealed non-activated areas. Deactivated regions were prominent with the motor extent threshold, but not close to the activated areas of the motor cortex. In the frequentist statistic both expert observer thresholds revealed similar activation in the right hand motor cortex and overall pronounced deactivated regions.

## Discussion

We introduced a novel approach for calculating Bayesian statistical maps of task-related BOLD activity in pre-operative fMRI. The Bayesian analysis permits identification of brain areas that are explicitly activated, deactivated, non-activated and areas with low-confidence, unlike conventional frequentist statistical analysis that only identifies activated and deactivated brain areas. We developed a new automatic estimation method for the effect size threshold required for the Bayesian analysis. We tested the approach in a group of healthy, awake volunteers in a 3T pre-operative scanner.

We measured fMRI activation due to a passive movement paradigm. We calibrated and tested for self-consistency the Bayesian analysis by comparison with our local practice based on frequentist statistical inference incorporating expert clinical judgment. Since definition of an effect size threshold is a central requirement of Bayesian analysis, we devised a method for operator-independent determination of a clinically-meaningful effect size threshold based upon the acquired fMRI data for two clinical scenarios: activation loci and activation area extent. The method relates the top 0.1% positive BOLD effect amplitude excursions observed in the data to the clinically meaningful minimal BOLD response amplitudes as judged by expert observers. In this study the maximal and minimal BOLD response amplitudes were found to be related by a simple factor that depended on the clinical objective. If activation loci were targeted, the relative factor was 0.497; if the activation extent was of interest, the factor was 0.144.

We tested the Bayesian analysis approach and automated γ estimation for self-consistency using data from a conventional 3T pre-operative scanner, specifically by comparing frequentist and Bayesian analysis. Comparing Bayesian versus frequentist analysis results, similar activation patterns were found in seven of 10 volunteers for the activation loci and similar motor area extent for all volunteers.

The analysis showed the expected surgically-relevant motor loci, motor area extent and somatosensory brain areas, except for the data of volunteers 9 and 10 that were categorized as outliers. These data showed implausible small effect sizes and activations. The sensitivity was high for 6 out of 10 volunteers for the motor localization and further overall increased for the motor extent. The false discovery rate was generally low.

The choice for frequentist analysis as reference is based on the fact that clinicians are familiar with the commonly used frequentist statistics for fMRI analysis and therefore we resort to this method as we have established in-house expert knowledge. Furthermore, for the healthy subjects scanned, electro-cortical mapping as an alternative independent reference was not available, and the representative patient data was obtained in advance of surgery so electro-cortical mapping or confirmatory surgical outcome data were also unavailable.

The impact of additional smoothing on the quantitative analysis by the interpolation process was negligible because we used a 7th order spline interpolation with high SNR (Thévenaz et al., 2000). The analysis on the upscale maps was chosen since it reflects the surgical workflow in which the fMRI results are presented on a high resolution structural scan.

An alternative Bayesian approach for estimating non-activated regions for surgical planning has been presented by Johnson et al. (2012). This alternative approach differs from our method in a number of ways. First, the method operates on Z-scores from a previous GLM analysis, rather than by direct Bayesian estimation of the parameters of a GLM as in our approach. Second, the prior distribution is based on a Potts model with Dirichlet process priors, rather than the spatial Gaussian priors in our approach. Third, the method has been applied to data from only a single subject, as compared to the 11 subjects in our study. Furthermore, the (implicit) effect size is estimated by the algorithm and not based on a calibration framework informed by clinical expert knowledge.

Our novel estimation of the minimal effect size is automatic and operator independent. It is calibrated with respect to the current expert-knowledge based clinical best judgment. As a caveat, care must be taken to exclude non-brain regions showing artefactual apparently high activation, e.g., in the orbits and veins, in applying this automated method (Turner, 2002), since these data will bias the effect size model. We overcame this using masks derived from brain tissue segmentation with SPM12b.

We postulate that our model is widely applicable to Bayesian fMRI analysis, although this requires validation in further studies using alternative paradigms, e.g., visual stimulation, active motor tasks or tactile stimulation. These studies will also address the potential circularity in the present study, which used the same data for both calibration and self-consistency test at 3T. In the worst-case scenario, the circularity may indicate validity of an incorrect effect size model, leading to under- or overestimation of the BOLD activation. We note that the effect size model was validated on the independent 1.5T dataset, avoiding circularity. We recommend further validation on independent datasets under different conditions and at different field strengths.

We understand further that the use of fMRI and frequentist analysis as the reference is a potential limitation. Future studies may compare the results with the gold standard intrasurgical electrocortical stimulation mapping (ESM).

Since the calibration reflects our workflow for presurgical planning, it might not be the calibration of choice for other sites. Nevertheless, the calibration is based on commonly used frequentist statistics and can be adapted to match different threshold procedures for presurgical fMRI at other sites. As an alternative to calibration, function arterial spin labeling techniques (Raoult et al., 2011; Vincent et al., 2013) or calibrated BOLD (Leontiev and Buxton, 2007) could be considered if available at the site and suitable for the patients.

We explored two strategies for thresholding fMRI maps using the motor extent and the motor loci. While the maps based on the motor extent calibration include small and potentially borderline activated areas, the motor loci threshold maps include mainly the central primary focus of the activation, potentially obscuring lower level activations. Thus the methods are expected to yield different, complementary maps. As a pragmatic solution for clinical interpretation we suggest the possibility of producing combined maps exploiting both thresholds.

The passive movement used here was designed to be readily performed by a trained operator, with no reliance on mechanical and electrical devices, to facilitate clinical and especially intra-operative implementation in anesthetized subjects. It has been shown recently that passive motion is equally reliable as an active finger task (Blatow et al., 2011). However, a potential drawback of this method is that operator-dependent variations of passive stimulation frequency and amplitude could affect the neuronal and BOLD responses and hence alter the activation pattern. It is possible that more consistent results may be achieved using a metronome temporal cue for the operator action or simple mechanical devices enforcing consistent guided motion of the fingers. It is, however, possible fully device-guided finger motion may also lead to decreased BOLD activation, as recently shown for certain tactile stimuli (van der Zwaag et al., 2013).

The Bayesian analysis of the 1.5T intra-operative scanner data showed activation patterns that were less clear but similarly located compared with the 3T data. In contrast, frequentist analysis revealed almost no active regions for the 1.5T scanner data. The 1.5T intra-operative scanner results showed generally lower significance and less certain localization information than the 3T results.

The quantitative cluster analysis revealed an improved sensitivity for the 1.5T Bayesian analysis results compared with the 1.5T frequentist results. This could be explained by the improved spatial SNR of the data due to the additional local smoothing of the Bayesian algorithm. The higher FDR rates are prominent in subjects with small activated clusters, increasing the impact of FP in the denominator.

While the benefits of our proposed fMRI analysis methods for pre-operative guidance are conceptually straightforward, advanced use for intra-operative guidance is more contentious. Despite our results showing that it is possible to acquire fMRI with the surgery head coil, the benefit of ifMRI as a tool for surgical re-planning during a craniotomy remains to be determined. The benefit and a potential improved patient outcome needs to be assessed and compared with alternative methods such as electrocortical stimulation mapping (Berger and Rostomily, 1997). Future work at our center and elsewhere will explore ifMRI as an additional data source to our routinely performed intra-operative structural imaging to update neuro-navigation as procedures progress.

We also note that the direct use of the 1.5T system PPMs (from ifMRI) without the PPMs from the 3T pre-operative scanner may be problematic, since the activity status of large brain regions could not be classified on the intra-operative scanner data. Hence, where possible it may be helpful to use pre- and intra-operative fMRI results in combination.

Several issues remain to be addressed before initial application of our method for ifMRI during neurosurgery, including the potential effects of anesthetic level on the BOLD signal, and other factors that may affect BOLD signal detection during craniotomy and dural incision, e.g., brain pulsation, air cavities, and blood coagulation (Gasser et al., 2005). In our study the effect-size model was developed in healthy awake volunteers; across the spectrum of neurological disease the effect size model may be inaccurate and require further validation. For example, the model could not account for hypothetical pathological non-linear BOLD response changes, i.e., a disease condition affecting only the most highly activated voxels rather than causing a global scaling of the BOLD response. Thus pathology in the highly activated voxels would bias estimation of the clinically-meaningful minimal effect-size threshold yielding inaccurate brain activity maps.

The 8-channel intra-surgery coil used here provides sufficient quality for structural images and is successfully used for routine iMRI neuro-navigation. However an improved-sensitivity coil design may be beneficial, since the multi-channel receive coil elements of our existing unit cover only the anterior and posterior cranial regions, with no coil elements located adjacent to the vertex near the motor cortex. Hence we presume that the regionally reduced coil sensitivity contributed in part to the lower activation significances and more extensive unclassified regions in the intra-operative scanner data.

We tested our novel approach for thresholding Bayesian statistical maps of task-related BOLD activity on a brain tumor patient. We acquired our passive movement paradigm on the 3T presurgical scanner and derived activation maps for the motor localization and motor extent using our Bayesian statistics framework. For the Bayesian result maps we used our novel threshold method. The frequentist statistics results were based on the expert observers thresholds. Bayesian and frequentist statistics revealed activation in the motor cortex for both clinical scenarios (targeting the extent or location of the motor cortex), as well as deactivation in various areas. Bayesian statistics revealed additionally non-activated and low-confidence areas.

In this clinical case the motor-strip BOLD activation was not expected to suffer from tumor-related abnormal vessel blood flow and lack of auto-regulation in the tumor, as the lesion is inferior to the hand knob. However, in future studies a perfusion map to exclude abnormal vascularization, not part of the current study protocol, might be helpful.

The deactivated areas are neither in the primary motor cortex nor in the tumor and are not directly adjacent to the activated area. Therefore we consider those areas to be correctly identified and not be paradoxically negative BOLD (Fujiwara et al., 2004). However in patients with tumors in eloquent brain areas this might apply and therefore activated and deactivated areas must be assessed in combination (Fujiwara et al., 2004).

We can further exclude influences on the BOLD response by medication, e.g., acetazolamide (Brown et al., 2003) and impaired attention. However, we can not exclude that the patient age (58 years) had an impact on the image intensity (Chen et al., 2008). To cope with potential abnormal hemodynamic response functions in tumors patients, dispersion derivatives (Friston et al., 1998) or more sophisticated approaches (Chaari et al., 2013; Woolrich et al., 2004b) could be used in future applications of our method.

As an alternative approach to passive fMRI or direct cortical stimulation, resting state fMRI has recently been proven to offer similar sensitivity and specificity without requiring the patient's active cooperation (Qiu et al., 2014). However the authors did not address whether this still pertains when a lesion affects the motor cortex, or how resting state fMRI performs under tumor-induced brain plasticity. Alternative, topological analysis (Due-Tonnessen et al., 2014) also cannot detect tumor induced brain plasticity, because only structural brain parameters are considered, although this method is reliable where the central sulcus is not, or is only moderately distorted by the lesion (Due-Tonnessen et al., 2014).

General application in patients may also pose other new challenges: cerebral blood flow and BOLD response may be affected by tumor pathology or other malformations. One approach to reduce the potential impact of physiological noise is advanced correction (Josephs et al., 1997; Hutton et al., 2011) incorporating physiological monitoring signals. Patient compliance may also differ significantly (claustrophobia, impaired attention, …), although the passive motion paradigm is expected to be minimally affected by this.

## Conclusion

We implemented, calibrated and tested for self-consistency a Bayesian fMRI analysis framework using passive motor task data from a conventional 3T MRI scanner and a 1.5T intra-operative MRI scanner. In comparison to frequentist analysis we demonstrated that Bayesian fMRI analysis yields identification of activated, deactivated and non-activated brain regions, important for guiding brain tissue resection avoiding functionally eloquent brain areas. Additionally, the ability to explicitly identify remaining regions, which could not be classified in the Bayesian analysis, is also important, as these represent the limits of the information available from the fMRI experiment. These maps will further empower surgeons to address the practical problem of ambiguity in fMRI results, which may be more pronounced in intra-operative MRI scanners due to generally lower static magnetic field strengths and RF coil placement restrictions.

### Conflict of interest statement

The Wellcome Trust Centre for Neuroimaging at UCL has an institutional research agreement with and receives support from Siemens Healthcare. Craig Buckley is employee of Siemens Healthcare UK. The funders had no role in study design, data collection and analysis, decision to publish, or preparation of the manuscript. The authors declare that the research was conducted in the absence of any commercial or financial relationships that could be construed as a potential conflict of interest.

## Footnotes

^1^In house software, under development and not currently published.

^2^http://www.fil.ion.ucl.ac.uk/spm/, 12th January 2015.

^3^http://fsl.fmrib.ox.ac.uk/fsl/fslwiki/, 12th January 2015.

^4^http://www.mathworks.com, 12th January 2015.

^5^http://www.debian.org, 12th January 2015.

^6^The mean processing time for the Bayesian analysis was 11 min with a standard deviation of 1:15 min.

^7^MATLAB function “bwlabeln” with connectivity 18.

## Abbreviations

CSB: cluster size in the Bayesian analysis
CSF: cluster size in the frequentist analysis
γ: effect size threshold
γ_loc_: minimal BOLD response effect size for activation loci
γ_ex_: minimal BOLD response effect size for activation extent
LBT: Log Bayes factor thresholdl
t_loc_: observer selected t-threshold for activation loci
t_ex_: observer selected t-threshold for activation extent
J: Jaccard index.
